# An evolutionarily significant unicellular strategy in response to starvation in *Dictyostelium* social amoebae

**DOI:** 10.12688/f1000research.4218.2

**Published:** 2014-12-05

**Authors:** Darja Dubravcic, Minus van Baalen, Clément Nizak

**Affiliations:** 1 CNRS, LIPHY, F-38000 Grenoble, France; 2 Laboratory of Ecology and Evolution, CNRS UMR7625, Ecole Normale Supérieure, Université Pierre et Marie Curie, Paris Universitas, CNRS, Paris, France; 3 Laboratory of Biochemistry, UMR 8231 ESPCI ParisTech/CNRS, PSL Research University, Paris, France

## Abstract

The social amoeba *Dictyostelium discoideum *is widely studied for its multicellular development program as a response to starvation. Aggregates of up to 10 ^6^ cells form fruiting bodies containing (i) dormant spores (~80%) that can persist for months in the absence of nutrients, and (ii) dead stalk cells (~20%) that promote the dispersion of the spores towards nutrient-rich areas.

It is often overlooked that not all cells aggregate upon starvation. Using a new quantitative approach based on time-lapse fluorescence microscopy and a low ratio of reporting cells, we have quantified this fraction of non-aggregating cells. In realistic starvation conditions, up to 15% of cells do not aggregate, which makes this third cell fate a significant component of the population-level response of social amoebae to starvation. Non-aggregating cells have an advantage over cells in aggregates since they resume growth earlier upon arrival of new nutrients, but have a shorter lifespan under prolonged starvation. We find that phenotypic heterogeneities linked to cell nutritional state bias the representation of cells in the aggregating vs. non-aggregating fractions, and thus affect population partitioning. Next, we report that the fraction of non-aggregating cells depends on genetic factors that regulate the timing of starvation, signal sensing efficiency and aggregation efficiency. In addition, interactions between clones in mixtures of non-isogenic cells affect the partitioning of each clone into both fractions. We further build a numerical model to test the evolutionary significance of the non-aggregating cell fraction. The partitioning of cells into aggregating and non-aggregating fractions is optimal in fluctuating environments with an unpredictable duration of starvation periods. Our study highlights the unicellular component of the response of social amoebae to starvation, and thus extends its evolutionary and ecological framework.

## Introduction

Every organism has a set of optimal conditions that maximizes its fitness (growth, reproduction and survival). Yet, environments typically deviate from these conditions. In some cases individuals can adapt to changes by sensing the environment and modifying their phenotypes accordingly, which is known as phenotypic plasticity ^1^. However, if the sensing mechanism is too costly, phenotypic plasticity may not be optimal even in the presence of environmental variation. Differentiation on a stochastic basis into different phenotypic states adapted to different environments, also known as risk spreading or bet hedging, has also been proposed as an adaptation to environmental variation ^2– 6^. Dormant states have often been described as such bet hedging strategies. Examples include plant seed dormancy ^7^, arthropod diapauses ^8^ and bacterial sporulation ^9^. For entering and exiting the dormant state, cells or organisms depend on environmental cues. Yet, these cues are not always reliable indicators of the future environment. Therefore, in such unpredictable environments it pays off for a plant, for instance, to have its seeds germinating stochastically at different time scales to insure that at least some of them will germinate at the time that is beneficial for its growth ^7^.

Here we focus on the dormancy of the cellular slime mold *Dictyostelium discoideum* as an adaptation to nutritional stress. *D. discoideum* amoebae live in soil where they feed on bacteria and divide mitotically. When starved, cells enter into the dormant social phase of the life cycle. Up to 10 ^6^ cells aggregate to form a multicellular organism that goes through a “slug” stage followed by the formation of a fruiting body. The slug is a motile, chemotactic and phototactic worm-like structure that senses and moves towards environments that are favorable for dispersion, germination and cell proliferation. The fruiting body is a sessile mushroom-like structure with the spore mass sitting on top of a stalk. Dormant spores can survive for months in the absence of food, and germinate into single cells upon dispersion towards nutritive areas. The stalk lifts the spores from the ground, which helps spore dispersion. Cells in the stalk, which represent ~20% of the total cell population, die owing to the metabolic cost of making up the stalk ^10^.

Its social behavior has made *D. discoideum* a very popular system for studying altruism, cheating and cooperation ^11, 12^, but not all aspects of its population-level adaptation to stress have been studied. Our main motivation was to study a previously known but neglected fact that not all cells aggregate upon starvation ^13^. We have thus revisited the *D. discoideum* population-level response to nutritional stress by focusing on the aggregation stage. Incomplete aggregation may have significant evolutionary consequences. Aggregation is costly due to the death of stalk-forming cells and the arrest of cell division during fruiting body formation, which is an irreversible process ^14^. Cells that do not aggregate do not pay these costs and may have the advantage of resuming growth immediately upon arrival of new nutrients. If conditions improve quickly, non-aggregating cells thus may have an important adaptive advantage.

In this study we present the first attempt to describe the *D. discoideum* response to starvation as a functional partitioning into two states: aggregating and non-aggregating. We focus on two major points: (i) establishing the phenotypic and genotypic sources of population partitioning and (ii) assessing the evolutionary significance of such partitioning. In microbial systems, cell states such as cell cycle phase, nutritional state or age are sources of phenotypic heterogeneities ^9, 15^. Besides, different genetic backgrounds could give rise to different degrees of heterogeneity, giving insights into underlying molecular mechanisms. Here we develop a new technique based on quantitative live cell microscopy to analyze the effects of cell nutritional state, genetic background and environmental organization on population partitioning between aggregating and non-aggregating cells. In addition, we propose a model based on experimentally determined parameters to illustrate the potential evolutionary significance of population partitioning in fluctuating environments.

## Materials and methods

### *D. discoideum* strains and culture

*D. discoideum* axenic strains used in the study were AX3 (Dictybase ID: DBS0235545), DH1 (Dictybase ID: DBS0302388), *phg2* (Dictybase ID: DBS0302388), *pdsA* (Dictybase ID: DBS0237030), and *carA* (Dictybase ID: DBS0236438). All the strains were cultured in autoclaved HL5 medium (per L, 5 g proteose peptone, 5 g thiotone E peptone, 5 g yeast extract, from USBIO, 10 g glucose, 0.35 g Na _2_HPO _4_*7H _2_O, 0.35 g KH _2_PO _4_ from Sigma-Aldrich, pH=6.7) at 22°C if not mentioned otherwise. In experiments on nutritional effect we used: FM minimal medium (Formedium), NS (per L, 15.2 g peptone, 7.6 g yeast extract, from USBIO, 5mg Na _2_HPO _4_, 5mg KH _2_PO _4_, from Sigma-Aldrich, pH=6.7) and NS with 85mM glucose (Sigma-Aldrich) added after autoclaving ^15^. The bacterial species used as the nutritional source in our study was *Klebsiella aerogenes*. Heat killed bacterial cultures were prepared by centrifuging 50mL of overnight LB cultures at 4°C, 5000 g for 10min and diluting the pellet in 1mL KK2 buffer (per L, 22 g KH _2_PO _4_, 7.0 g K _2_HPO _4_, Sigma-Aldrich). The suspension was incubated for 20min at 80°C and stored at -20°C.

### GFP and RFP-expressing cell lines

GFP and RFP-expressing cell lines were obtained by transforming cells with pTX-GFP (Dictybase ID: 11) ^17^ or pTX-RFP (Dictybase ID: 112) plasmids using a standard electroporation procedure. Cells were grown in 75cm ^2^ flasks until dense but not confluent (usually 1 day before confluency). The medium was changed 4–6h before transformation. For transformation cells were re-suspended in 10mL of ice-cold HL5 and kept on ice for 30min. Cells were centrifuged for 5min, 500 g at 4°C. Supernatant was re-suspended in 800μl of electroporation buffer and transferred into ice cold 4mm electroporation cuvettes containing 30μg of plasmid DNA. Cells were electroporated at 0.85 kV and 25 mF twice, waiting for 5 s between pulses. Cells were transferred from the cuvette to 75cm ^2^ flask with HL5. The next day, transformants were selected with 5μg/ml G418 (Sigma-Aldrich). The concentration of G418 was gradually increased to 20μg/ml G418 over 1–2 weeks. Transformed strains were maintained at this concentration of G418, yielding GFP and RFP-expressing cell lines that were analyzed by flow cytometry on a Becton-Dickinson LSRII analyzer to confirm their unimodal cellular fluorescence distribution (>99% of fluorescent cells upon analysis of 10 ^6^ cells, see Supplementary Figure S6).

### Starvation protocols

Cells were subjected to two different starvation conditions: sudden and gradual starvation. For each condition measurement was repeated 4–11 times (see Raw data for further details, each measurement is an independent experiment).

***Sudden starvation:*** If not mentioned otherwise, sudden starvation was used as a standard plating protocol: When confluent the cell medium with antibiotics was replaced with an antibiotic free medium. After 4–6h cells were washed out of the nutrient medium and centrifuged in KK2 buffer at 500g for 5 min. The pellet was re-suspended in KK2 buffer to the concentration of 1×10 ^5^ cells/μL. For the density dependent aggregation experiment cells were re-suspended to the concentration of 1×10 ^3^, 1×10 ^4^, 5×10 ^4^, 1×10 ^5^ or 5–7.5×10 ^5^ cells/μL. Green and red fluorescent cells were mixed in ratios indicated in Image analysis section. 30μl of suspension was plated on 6cm plates filled with 2mL of 2% Phytagel (Sigma-Aldrich) as previously described ^18^. In the case of pairwise mixtures, strains grown in different media or genetically different strains, the ratio of two strains was 1:1.

***Gradual starvation*** was induced in liquid cultures and on bacterial plates.

***Gradual starvation in liquid:*** the cells were collected 1–2 days after reaching confluency in HL5. Cell washing and plating was done as in sudden starvation experiment described above.

***Gradual starvation on bacterial plates:*** another way of slowly starving the cells is to plate them with bacteria and to let them deplete the food source as in natural conditions. Two types of plating were done: homogenous and heterogeneous plating. In both cases RFP-expressing AX3 and GFP-expressing AX3 cells were grown in HL5 medium with 20μg/mL G418. When confluent, cells were re-suspended in KK2 buffer and centrifuged at 500 g for 5min. The cell pellet was re-suspended in KK2 to the concentration of 1×10 ^5^ cells/μL. Green and red fluorescent cells were mixed in ratios indicated in Image analysis section. For heterogeneous plating 200μL of heat-killed bacteria was mixed with 100μl of cell suspension. The mixture was spread on a 6cm plate with 2mL of 2% Phytagel (Sigma-Aldrich). This gave rise to heterogeneous distribution of cells and bacteria ( Supplementary Figure S2). For homogenous plating 200μl of heat-killed bacteria were mixed with 100μl of cell suspension. A 100μl drop was plated on a 6cm plate with 2ml of 2% Phytagel and let to dry under the sterile hood. This gave a very homogeneous cell distribution ( Supplementary Figure S2). In both cases, cells fed for ~8h on heat-killed bacteria before the beginning of starvation, and thus divided at most twice after plating. The density of cells at the onset of starvation (measured via a similar method as the one for measuring the non-aggregating cell fraction, see below) was comparable to that of cells processed according to the sudden starvation protocol.

### Time-lapse microscopy

The 6cm diameter Petri dish was imaged on an automated inverted microscope setup duplicated from a previous study ^19^. The setup was made of: OlympusIX70 inverted microscope, Photometrics CoolSNAP HQ ^2^ CCD camera, Zeiss HBO 100 microscope illuminating system, Thorlabs SH05 shutter, Thorlabs TSC001 shutter controller, and 2.5×-5×-10×-20× objectives (5× was used for all experiments shown here). Images were acquired in WinView/32 and the whole setup was controlled by custom-made Visual Basic software. The setup allows Petri dish scanning at regular time intervals (typically 1h), with phase contrast and fluorescence image acquisition at all time points (at 100ms and 1s exposure times respectively). A mosaic image is reconstructed by combining all the images of contiguous areas of the Petri dish at a given time point by a custom-made macro using ImageJ software ( http://rsbweb.nih.gov/ij/).

### Image analysis

**Figure 1.**
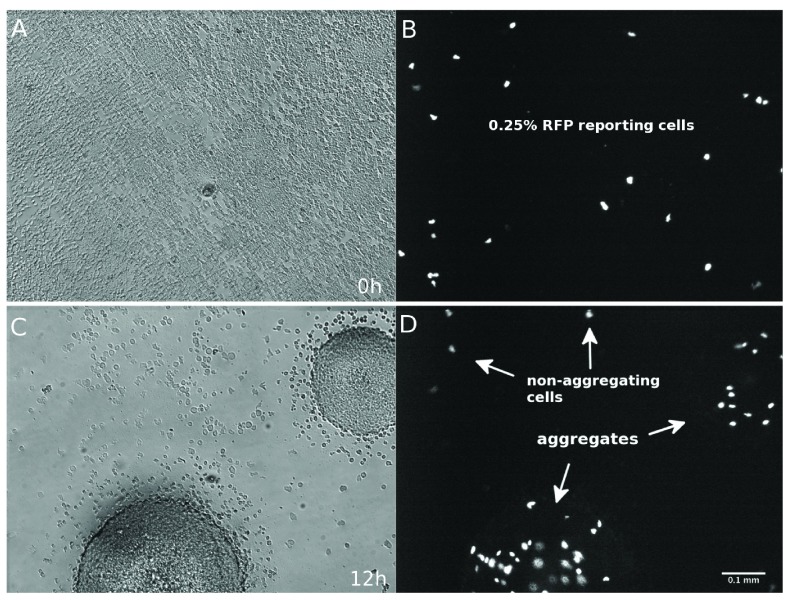
Upon starvation, a *D. discoideum* population partitions into aggregating and non-aggregating cells. AX3 cells were plated on nutrient free-agar and imaged before ( **A**, **B**) and after ( **C**, **D**) aggregation. **A** and **C** are phase contrast images, **B** and **D** are red fluorescence images. In **B** and **D**, 0.25% of AX3 RFP cells appear as single dots within a population of AX3 GFP cells. The percentage of non-aggregating cells was estimated as the ratio of dots counted outside aggregates after aggregation and dots counted before aggregation.

Mixing a small percentage of red fluorescent cells in a population of green fluorescent cells allowed us to get the image of single cells as single red fluorescent dots ( Figure 1). We also confirmed that the reciprocal mixing of a minority of GFP-expressing cells with a majority of RFP-expressing cells yields the same results. We optimized the red to green cell ratios depending on plated cell density. For experiments with 1×10 ^5^ and 5–7.5×10 ^5^ cells/μL, 0.25–0.5% of RFP cells were mixed with 99.5–99.75% GFP cells. For 1×10 ^4^ cells/μL 1% of RFP cells were used and for 1×10 ^3^ cells/μL 2% RFP cells were used. For pairwise mixtures the ratio was made as following: 50% of strain A in GFP was mixed with 49.75% of strain B in GFP and 0.25% of strain B in RFP in order to monitor the behavior of strain B in a A:B mixture. Images were acquired by time-lapse fluorescence microscopy. All the images were analyzed using ImageJ software ( http://rsbweb.nih.gov/ij/) using custom-made macros (see Data Set). The analysis consisted in counting fluorescent dots before and after aggregation. For each experiment 1000–10 000 dots/cells were monitored. Dead cells were excluded from counting by looking at cell displacement as an indicator of cell viability. Two fluorescent images taken 1–2h apart were overlapped and cells that showed no displacement were counted and subtracted from the overall non-aggregating population.

The density of red dots (RFP-expressing cells) was used to estimate cell density at the onset of starvation in all experiments. Cell density was comparable at the onset of starvation for all starvation protocols used.

### Spore germination

Spore formation was induced by separately plating AX3 RFP and AX3 GFP cells on nutrient-free Phytagel plates. Once fruiting bodies had formed spores were picked using 1ml pipette tips and re-suspended at high density in 5m liquid HL5 with 70μL of dead bacteria culture. Presence of bacteria helped to induce spore germination. When the culture of germinated spores reached confluency (15–20h after plating) cells were washed of bacteria in ice cold KK2 and plated according to the Sudden starvation protocol.

### Model

The model represents the *D. discoideum* life cycle with alternating growth and starvation periods of variable duration. During the growth phase the population grows according to a logistic Equation (1) with growth rate *λ* and carrying capacity *K = N*_max_,

$$\frac{\text{dN}}{\text{dt}}=\lambda N(1-\frac{N}{K})\;\;\;\;\;\;\;\;\;\;\;\;\;\;\;\;\;(1)$$

We assume that the growth phase lasts sufficiently long for the population to have reached maximum density *K* when the food eventually runs out and a starvation period *T* sets in. The population then splits into an aggregating ( *N*_agg_ = *αN*) and a non-aggregating ( *N*_non-agg_ = (1– *α*) *N*) fraction according to the aggregation factor *α*. Aggregating cells subsequently differentiate into spore and stalk cells with the proportion of spore cells given by sporulation efficiency *s*, so *N*_spores_ = *sN*_agg_. We assume the process of aggregation is very quick relative to the duration of the growth and starvation periods. During the starvation period spores are dormant; their growth and mortality rate are assumed to be zero. When conditions become favorable again, spores germinate with germination efficiency *g* and start dividing, but only after a fixed and non-negligible development time *D*. During the starvation period the non-aggregating cells do not divide and are subjected to mortality with instantaneous mortality rate *μ*, so that their dynamics are governed by

$$\frac{dN_{\text{non−agg}}}{dt}\;=-{\text{μN}}_{\text{non−agg}}.$$

The advantage that non-aggregating cells have is a head start when conditions improve, as spores produced by aggregating cells need time to develop from the point they irreversibly commit to fruiting body formation (20h) and to subsequently germinate (3h–6h). By the time the latter start growing, the descendants of the non-aggregating cells may have the opportunity to use up a sizable portion of the resources that have become available. Here, we assume that spore germination is limited by the remaining carrying capacity.

As a first step in understanding the relative benefits of aggregation and non-aggregation consider the fates of cells of either type at the moment starvation sets in. A non-aggregating cell stops reproducing but is subject to mortality so when conditions become favourable again, *T* time units later, it has a probability *e ^-μT^* of surviving the starvation period. Working out the fate of aggregating cells is simple: it has a probability *sg* of becoming a germinating spore when conditions improve. An aggregating cell thus has a fitness equivalent of

$$W_{\text{agg}}=sg.$$

As discussed, germination involves a time cost: during a time *D* its surviving non-aggregating competitors can start reproducing, giving the latter an extra reproduction bonus (a period of logistic growth), giving a fitness equivalent of

$$W_{\text{non}-\text{agg}}=e^{-\mu T}\frac{e^{\lambda D}}{1+\frac{n_{0}}{K}(e^{\lambda D}-1)},$$

where *n*_0_ is the number of surviving non-aggregating cells.

The expected fitness (descendants by the time conditions improve) of a cell that has a propensity *α* to aggregate can thus be expressed as

$$W=\alpha W_{\text{agg}}+(1–\alpha )W_{\text{non}-\text{agg}}.$$

This result suggests that (if the duration of the starvation period is fixed) it is either profitable to join an aggregation (if *W*_agg_ > *W*_non-agg_) or to stay solitary (if *W*_agg_ < *W*_non-agg_): a phenotype-switching strategy is not favored. However, this result does not take into account the frequency dependence that acts on the fitness of non-aggregating cells. That is, if many cells aggregate the number of surviving non-aggregating cells ( *n*_0_) will be low, boosting the profitability of remaining solitary. If many cells remain solitary, on the other hand, *n*_0_ will be high, reducing the profitability of remaining solitary. Whether this frequency dependence results in population heterogeneity cannot be stated right away and other methods are necessary. The same is true when the environment, and in particular the starvation period *T*, is variable and unpredictable.

In order to study potential benefits of producing both aggregating and non-aggregating cells, strains with different aggregation factors α were put in competition using a multistrain variant of the above-described model. The population is made of *i* strains each with α=0 (all cells aggregate), 0.1, 0.2, … 1 (none of the cells aggregate). All strains had the same growth rate *λ* = 0.38, mortality rate *μ* = 0.002 for t ≤ 168h (7 days), after 7 days all cells die, *μ* = 0, sporulation efficiency *s* = 0.8 and germination efficiency *g* = 0.63. All values are based on experimental measurements (Materials and Methods in Supplementary materials). Two-step mortality function is an approximation based on our unpublished results and previous studies ^20, 21^. The precise shape of this function had no significant effect on our main observations and conclusions. Competition was carried out in two types of conditions, either constant or varying starvation periods T. In the case of varying starvation periods, the duration of starvation was randomly chosen from a uniform distribution *U(x,y)* at the end of every growth period. Population size was taken as an estimate of strain fitness. At the end of every growth cycle, the number of alive and growing individuals *N(t)* is plotted. In the case of varying starvation periods, the geometric mean over 100 simulations is plotted.

**Figure 2.**
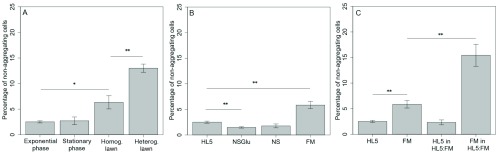
Starvation conditions, nutritional state and population partitioning. The percentage of non-aggregated cells (at initial density 3×10 ^6^ cells/cm ^2^) was measured for different initial cell states. **A**) Effect of starvation conditions. AX3 RFP and GFP cells were starved suddenly at exponential phase or at stationary phase, or gradually on homogenous bacterial lawns or on heterogeneous bacterial lawns. Gradually starved cells aggregate less than cells submitted to standard but less realistic sudden starvation protocols. **B**) Effect of nutritional state. AX3 cells were grown on HL5 rich medium, FM minimal medium, NS with 85mM Glucose (NS Glu) or NS medium, and subsequently plated on nutrient-free agar. Cells in the lowest nutritional state (FM) aggregate significantly less than cells fed with rich medium. **C**) Interactions between cells in different nutritional states. AX3 cells grown on HL5 or FM were plated either on their own or in 1:1 mixtures on nutrient-free agar (HL5inFM = HL5 cells monitored in 1:1 mixtures, and FMinHL5 = FM cells monitored in 1:1 mixtures). In mixtures with HL5-grown cells, FM-grown cells aggregate even less than on their own, while HL5-grown cells aggregate equally well in the presence of FM-grown cells as on their own. Note that the non-aggregating cell fraction of the global mixed population is higher than that of either pure population. Error bars represent +/- standard deviation (5≤n≤11). * represents p<0.05. ** represents p<0.01 (horizontal bars indicate the corresponding pairs of statistical samples).

**Figure 3.**
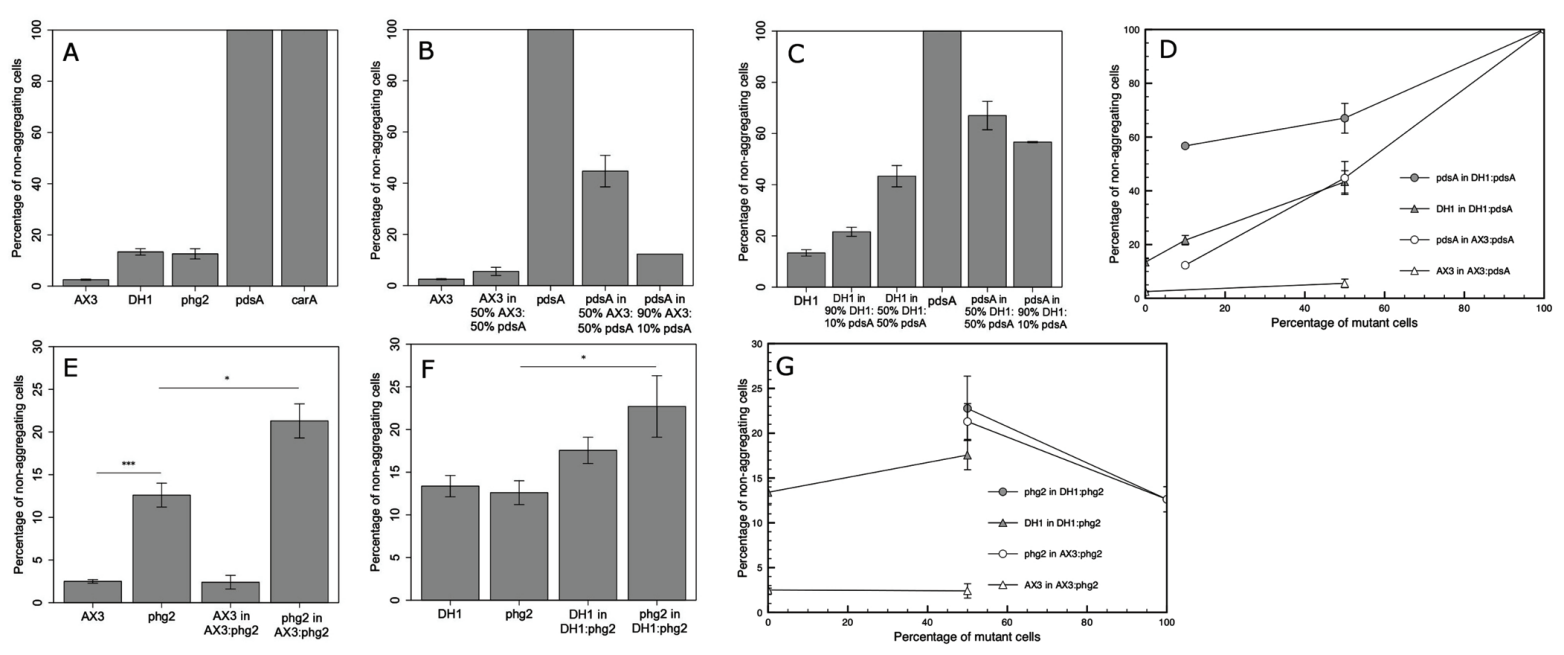
Genetic factors affect population partitioning. The percentage of non-aggregated cells (at initial density 3×10 ^6^ cells/cm ^2^) was measured for genetically different wild-type strains (AX3 and DH1) and single-gene mutants ( *phg2*, *pdsA*, *carA*) alone ( **A**), and in mixtures between wild-type and single-gene mutant strains: mixtures of *pdsA* with AX3 ( **B**) or DH1 ( **C**), and mixtures of *phg2* with AX3 ( **E**) or DH1 ( **F**)., varying the percentage of mutant cells in mixtures. Wild-type DH1 cells aggregate less than wild-type AX3 cells ( **A**). *phg2* mutant cells aggregate as well as their parent DH1 strain cells, while *pdsA* and *carA* cells do not aggregate on their own ( **A**). The presence of AX3 or DH1 cells rescues *pdsA* cell aggregation ( **B**– **D**). In turn, DH1 cells aggregate less than on their own when increasing the percentage of *pdsA* cells in DH1: *pdsA* mixtures, while AX3 cells aggregate as well as on their own in the presence of *pdsA* cells ( **B**– **D**). *phg2* cells aggregate less than on their own in the presence of AX3 or DH1 cells. DH1 cells aggregate less than on their own in DH1: *phg2* mixtures, while AX3 cells aggregate as well as on their own in the presence of *phg2* cells ( **E**– **G**). The non-aggregating cell fraction of the global mixed DH1: *phg2* or AX3: *phg2* population is higher than that of either pure populations, respectively DH1 and *phg2*, or AX3 and *phg2*. Overall, cell genotype determines the fraction of aggregating cells, and cells of different genotypes affect each other’s non-aggregating cell fraction in mixtures. Error bars represent +/- standard deviation. * represents p<0.05 (2≤n≤9). *** represents p<0.001 (horizontal bars indicate the corresponding pairs of statistical samples).

**Figure 4.**
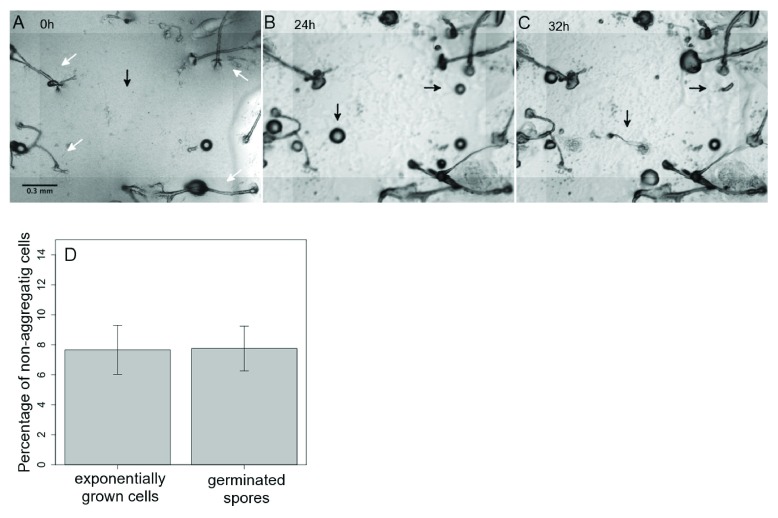
Cells switch between aggregating and non-aggregating fates. **A**– **C** Non-aggregating cells have not stably lost the ability to aggregate. **A**) After the completion of aggregation and formation of fruiting bodies (white arrows), bacteria were added to areas with non-aggregating cells (black arrows). Non-aggregating cells grow and divide on fresh nutrients (see Figure 5). Once bacteria are consumed, the descendants of non-aggregating cells aggregate ( **B**) and develop into a fruiting body ( **C**). **D**) A population of germinated spores re-partitions into aggregating and non-aggregating cells upon starvation. A population of spores was germinated and grown on bacteria for 3–5 cell divisions. When this population is plated on a nutrient-free substrate it partitions into aggregating and non-aggregating cells with the same proportions as populations of exponentially growing cells submitted to starvation. Error bars represent +/- standard deviation (4≤n≤7).

**Figure S1.**
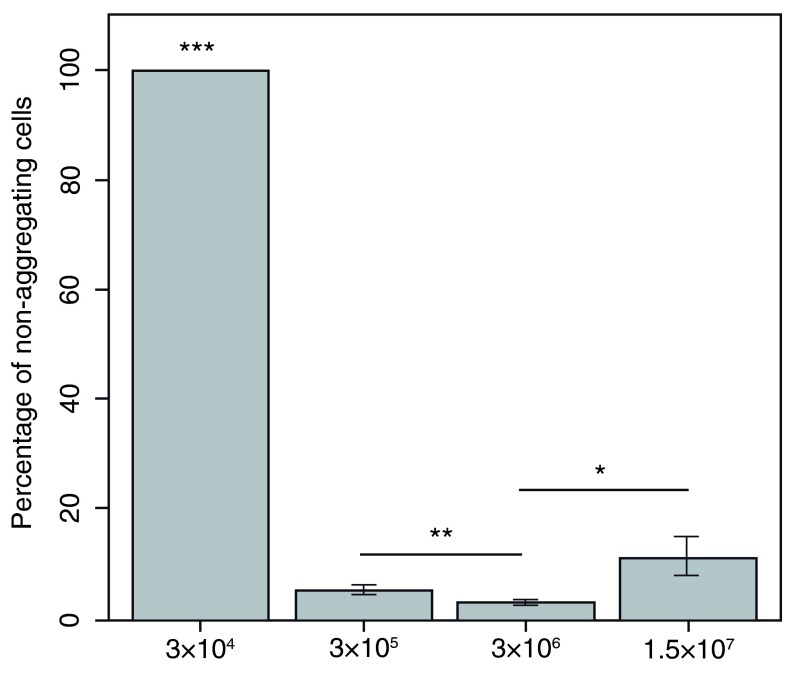
Effect of cell density on the non-aggregating cell fraction. AX3 cells were plated at initial densities 3×10 ^4^, 3×10 ^5^, 3×10 ^6^ and 1.5×10 ^7^ cells/cm ^2^ according to the sudden starvation protocol. Standard plating protocols mainly use 3×10 ^6^ cells/cm ^2^, which gave the lowest non-aggregating cell fraction. We plated cells at 3×10 ^6^ cells/cm ^2^ density in all further experiments. Error bars represent +/- standard deviation (4≤n≤10). * represents p<0.05. ** represents p<0.01. *** represents p<0.001 (horizontal bars indicate the corresponding pairs of statistical samples).

Aggregation vs. nonaggregation strategies in Dictyostelium discoideum amoebae in response to starvation stress: raw data Spreadsheet containing the data shown in Figure 2A. Spreadsheet containing the data shown in Figure 2B. Spreadsheet containing the data shown in Figure 2C. Spreadsheet containing the data shown in Figure 3A. Spreadsheet containing the data shown in Figure 3B. Spreadsheet containing the data shown in Figure 3C. Spreadsheet containing the data shown in Figure 3E. Spreadsheet containing the data shown in Figure 3F. Spreadsheet containing the data shown in Figure 4D. Spreadsheet containing the data shown in Sup. Figure S1. Spreadsheet containing the data shown in Sup. Figure S4. Spreadsheet containing the data shown in Sup. Figure S5. Macros is an archive containing the set of ImageJ macros used for image analysis that was used to produce the data shown in Figures 2, 3, 4, S1, S4 and S5. Code is the simulation code used in Figure 6.
Sup. Movie S1. Partitioning of Dictyostelium populations under starvation stress into aggregating vs non-aggregating cells. 0.25% or RFP-expressing AX3 cells mixed with 99.75% of GFP-expressing cells were plated according to the standard “sudden” starvation experimental protocol. After aggregates form, most single RFP-expressing cells are found in aggregates, and a minority of them are found outside of aggregates even though aggregating and non-aggregating cells were intermixed at the onset of starvation. Both phase contrast (left) and red fluorescent (right) images show that non-aggregating cells are alive and motile. Counting single RFP-cells before aggregation and cells that are outside of aggregates after aggregation provides a direct estimate of aggregating and non-aggregating cell numbers.
Sup. Movie S2. Non-aggregating cells have not stably lost the ability to aggregate. After the aggregation had finished and fruiting bodies started to form, fresh nutrients (dead bacteria) were added to the area with non-aggregating cells. Once bacteria have been consumed these cells aggregate and develop into a fruiting body.
Sup. Movie S3. Non-aggregating cells are capable of resuming growth immediately upon food arrival while aggregating cells are embedded in development. 18h after plating cells on nutrient-free agar, aggregating cells have formed slugs while non-aggregating cells are starving. Nutrition in form of dead bacteria was added at this point. Multicellular development goes on until the formation of fruiting bodies because cells in aggregates are irreversibly committed to development even in the presence of food. In contrast, nonaggregating cells feed on bacteria and divide several times. At the end of fruiting body formation, non-aggregating cells have already consumed most of the bacteria.

### Statistical analysis

Statistical analysis was performed in R. Significant difference between the samples was calculated using Welch two sample t test function in R (t.test(x,y)). To test among groups differences we used one-way ANOVA test in R, using oneway.test() function. When only p value is indicated it means that a t-test was performed, when p and F values are indicated ANOVA was performed. p<0.05 was considered significant.

## Results

**Figure S2.**
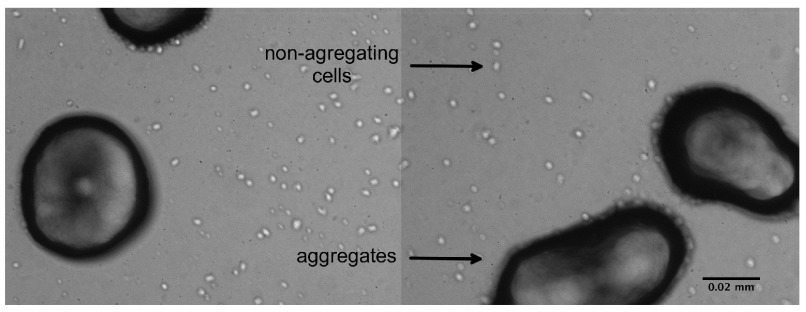
Non-aggregating cells of the NC 28.1 wild isolate. Cells were grown on bacteria and plated on nutrient-free agar according to the standard “sudden” starvation protocol. Wild isolate populations partition into aggregating and non-aggregating cells as laboratory strains do.

When we plated a population of genetically identical axenic wild-type AX3 cells of *D. discoideum* on nutrient-free substrates at a 10 ^4^–10 ^7^ cells/cm ^2^ density range ^22^, we observed that some cells aggregate while others remain outside of aggregates ( Figure 1, Supplementary Figure S1). A possible explanation is that the cells that did not aggregate are simply dead cells. However, the observation that non-aggregating cells are actively moving live cells that are intermixed with aggregating cells at the onset of starvation (Movie S1 in the Data Set below) rules out this possibility. It could also be that these non-aggregating cells have acquired a mutation that prevents aggregation. As we will detail further, this possibility can be ruled out by showing that the progeny of spores are partially non-aggregating and reciprocally that the progeny of non-aggregating cells aggregate upon starvation. Another explanation may be that partial aggregation is an artifact of a laboratory-adapted axenic strain that is not found in natural isolates, but in Supplementary Figure S2 we show that similar partitioning is found in natural isolates. Partitioning into aggregating and non-aggregating cells is therefore a process that occurs in both axenic strains and isolates of social amoebae from the wild. The non-aggregating cells we report here are clearly distinct from cells left in slug traces ^23^ since the former never aggregate as we have shown in Movie S1. For the same reason, non-aggregating cells are also clearly distinct from the immune-like cells identified in a previous study ^24^. The motility of the non-aggregating single cells we observe also rules out the possibility that these cells are sporulating without aggregating, as in single cell encystation that has been reported for other *Dictyostelium* species but not so far in *D. discoideum* ^10^.

To quantitatively analyze this process, we have developed a technique to track single cell behavior at each time point of the life cycle. Inspired by studies of cell motion within aggregates ^25^, a small proportion (0.25%–2%) of RFP-expressing reporting cells was mixed with GFP-expressing cells, and RFP cells were tracked (see Materials and methods). In the red fluorescence image single RFP cells appear as single red dots surrounded by undistinguishable GFP cells ( Figure 1B). Since cell division ceases during starvation, tracking RFP-expressing single cells allowed us to determine the relative numbers of aggregating vs. non-aggregating cells, and thus to quantitatively describe the population partitioning into aggregating and non-aggregating cells. Previous techniques based on counting cells at the onset of starvation with a hemocytometer and germinating/colony-forming spores provide only indirect estimation of the numbers of stalk cells, non-aggregating cells, or non-germinating spores. In contrast, our strategy provides a direct estimation of the numbers of cells at the onset of starvation and aggregating vs. non-aggregating cells. Our automated microscopy setup is similar to the one used in a previous study of large scale population spatial structure at the single cell resolution ^19^. We scan and image by phase contrast and fluorescence microscopy an area of 5cm ^2^ every 10min for 24h, allowing us to record the dynamics of the response of large populations (millions of cells) at the single cell resolution.

### Phenotypic plasticity affects population partitioning

Using our set-up, we found that when cells of the AX3 wild-type axenic strain are grown in liquid rich medium (HL5) and subsequently plated on nutrient-free substrate, 2.51±0.6% of the population does not aggregate. This standard starvation protocol involves the sudden transition from exponential growth in rich medium to starvation on nutrient-free agar. However, in natural conditions starvation is probably much more gradual. We analyzed how different starvation processes can affect population partitioning ( Figure 2A) at the same cell density range at the onset of starvation. We compared (i) suddenly starved exponentially growing cells, (ii) starved stationary phase cells (1–2 days after confluency), and (iii) cells grown on bacterial plates that slowly deplete the food source, the latter being the most realistic starvation process with respect to natural conditions. While stationary phase cells show no significant difference compared to exponentially growing cells, cells feeding on a homogenous bacterial lawn (see Methods) and thus gradually starving showed a 3-fold increase in the proportion of non-aggregating cells, 6.3±3.17% (p=0.027).

**Figure S3.**
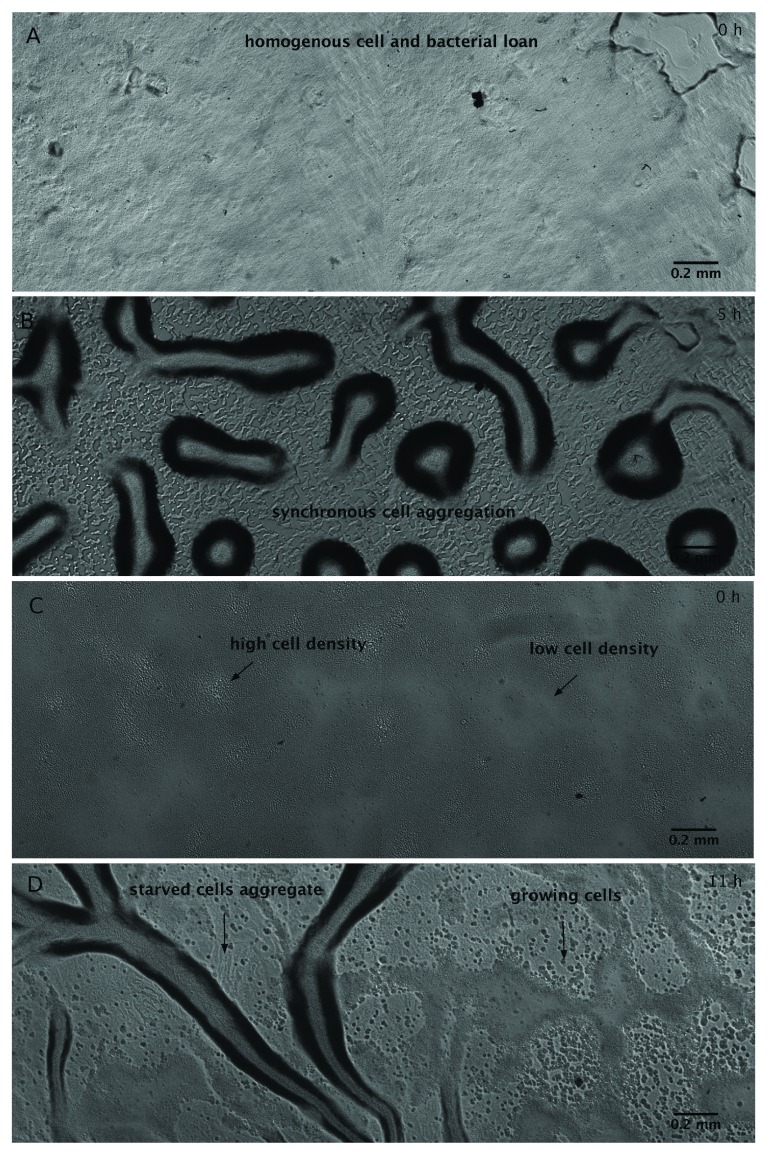
Cell aggregation on homogenous or heterogeneous bacterial lawns. AX3 cells were plated mixed with bacterial suspensions and plated on nutrient-free agar either homogenously ( **A**) or heterogeneously ( **C**). Homogenous plating yields a synchronous timing of starvation and aggregation over the whole plate ( **B**). Heterogeneous plating yields a non-uniform timing of starvation and aggregation, with aggregates forming in some areas while cells are growing in other areas ( **D**) of the same plate.

Gradual starvation on bacterial plates most likely increases heterogeneities in comparison with standard starvation protocols. We supposed that this was due to cell-to-cell differences in the timing of starvation. Some cells would start aggregating while others were not yet fully starved and therefore less sensitive (or not at all) to the aggregation signal. Increasing further heterogeneities during cell plating should thus increase further the non-aggregating cell fraction. This is indeed the case when a heterogeneous bacterial lawn (see Methods) is used as a food source, where the fraction of non-aggregating cells increases to 13%±1.79% (p=0.004). A possible explanation is that highly heterogeneous cell plating creates areas with different cell densities within a lawn of bacteria ( Supplementary Figure S3C, D). Areas with high cell densities deplete bacteria faster and start starving and aggregating quicker, while cells in low cell density areas still have nutrients surrounding them and are not sensitive to the aggregation signal when the former sense starvation. In homogenous bacterial lawns, cells and bacteria are evenly distributed favoring more homogenous and synchronous onset of starvation across the population ( Supplementary Figure S3). We hypothesized that differences at the onset of starvation result in a cell fate bias towards one phenotype or the other (as previously proposed in the case of stalk vs. spore differentiation in aggregates ^26^). To analyze these effects in the most reproducible and controllable manner, all following experiments were performed following the standard sudden starvation protocol (plating on nutrient-free agar) applied to cells grown in various well-defined conditions, with known genetic backgrounds, mixed at precise ratios and plated at controlled cell densities.

Nutritional state is known to affect whether a cell will become a spore or a stalk ^27^. Cells grown on rich medium (NS medium with 85mM glucose) are enriched in spores while cells grown in poorer medium (NS medium lacking glucose) are enriched in the stalk (which we have also observed, see Supplementary Figure S5). We thus asked whether nutritional state is a main determinant of the aggregating and non-aggregating dichotomy. We grew AX3 cells in media differing in nutrient content and analyzed whether they are differentially enriched in the non-aggregating state ( Figure 2B). Four different media that have extensively been used to culture *Dictyostelium* cells and manipulate their nutritional status were tested: on the one hand HL5 rich medium, FM minimal medium, on the other hand NS medium with 85mM glucose (NS Glu) and plain NS medium. AX3 cells grown on FM minimal medium showed a significant two-fold increase in the fraction of non-aggregating cells, 5.85±1.9% (p<0.01), with respect to HL5-grown cells (2.51±0.6%). In addition, cells grown on NS Glu showed a small but significant decrease in non-aggregating cells (1.47±0.31%, p<0.01) compared to HL5 grown cells (2.51±0.6%). However, we observed that cells grown in NS medium did not differ from cells grown in NS Glu in terms of non-aggregating cell fraction, making the role of glucose difficult to interpret. In this respect, it will be interesting to systematically modify a defined medium composition (such as that of FM medium) to point out particular types of nutrients, or ratios of nutrient concentrations, that influence population partitioning.

**Figure S4.**
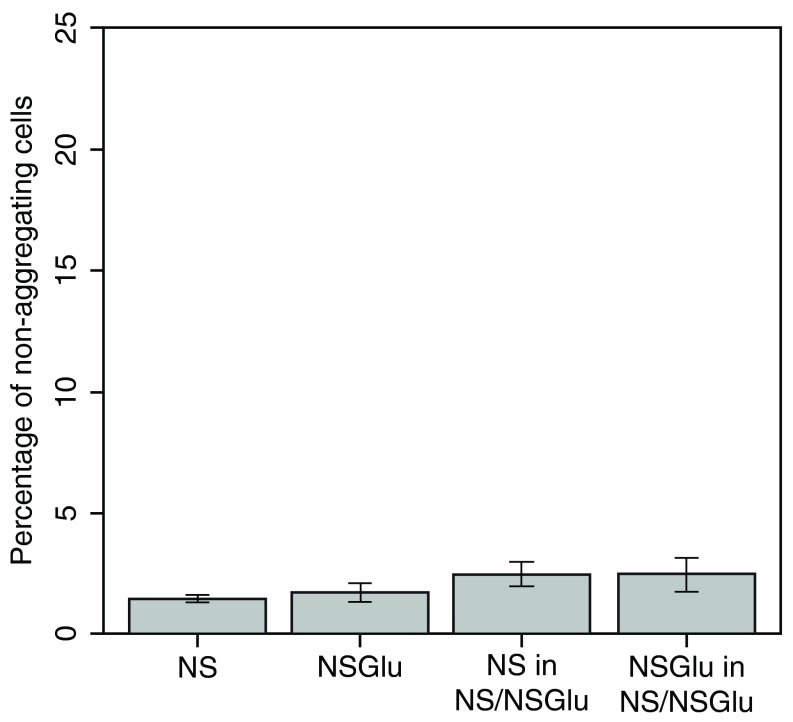
Effect of NS and NS Glu medium on non-aggregating cell fractions. Cells were grown either on NS and NS with 85mM glucose (NS Glu) medium. Cells grown on either medium show no differences with respect to the non-aggregating cell fraction when alone compared to when NS-grown and NSGlu-grown cells are mixed together. Error bars represent +/- standard deviation (6≤n≤8). A one-way ANOVA test yielded F=1.54, p=0.27.

**Figure S5.**
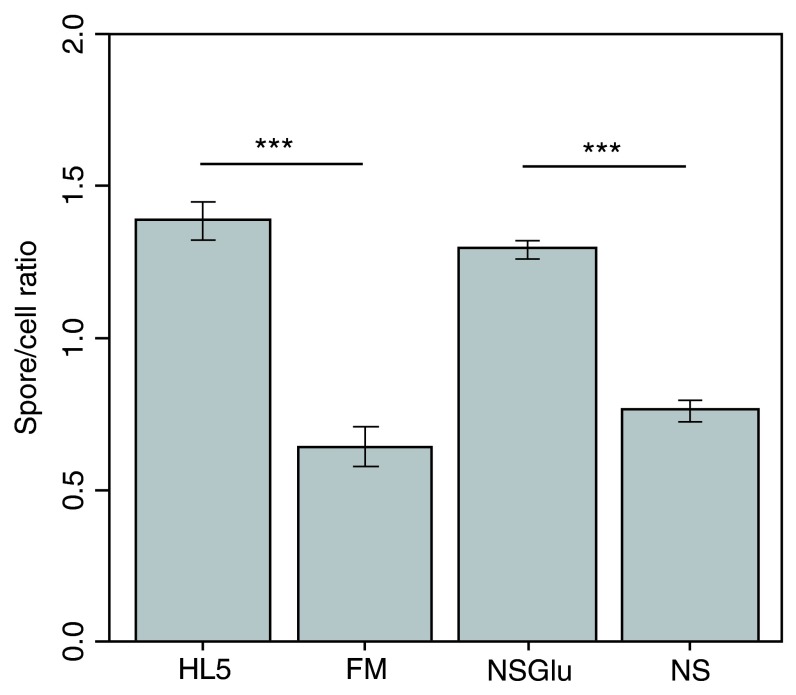
Nutritional state of the cells biases spore/stalk differentiation in aggregates. RFP-expressing AX3 and GFP-expressing AX3 cells were grown on one of 4 different media: HL5 (rich medium), FM (minimal medium), NS Glu (rich medium with glucose) and NS (rich medium without glucose). Cells grown on these media were mixed at 1:1 ratio for HL5:FM and NSGlu:NS mixtures. The ratio of spore percentage over plated cell percentage of each cell population in the 1:1 mixture indicates if one population is getting enriched into spores with respect to the other one. For spore/cells ratios = 1 both populations contribute equally to spores. If spore/cells ratio > 1, the population is enriched in spores, and if spore/cells ratio < 1, the population is underrepresented in the spores. We find that HL5-grown cells are over-represented in the spores in mixture with FM-grown-cells, FM-grown cells are underrepresented in the spores in mixture with HL5-grown cells. We confirm previously published data showing that NSGlu-grown cells are over-represented in mixture with NS-grown-cells, NS-grown cells are underrepresented in the spores in mixture with NSGlu-grown cells. Error bars represent +/- standard deviation (11≤n≤18). *** represents p<0.001 (horizontal bars indicate the corresponding pairs of statistical samples).

**Figure S6.**
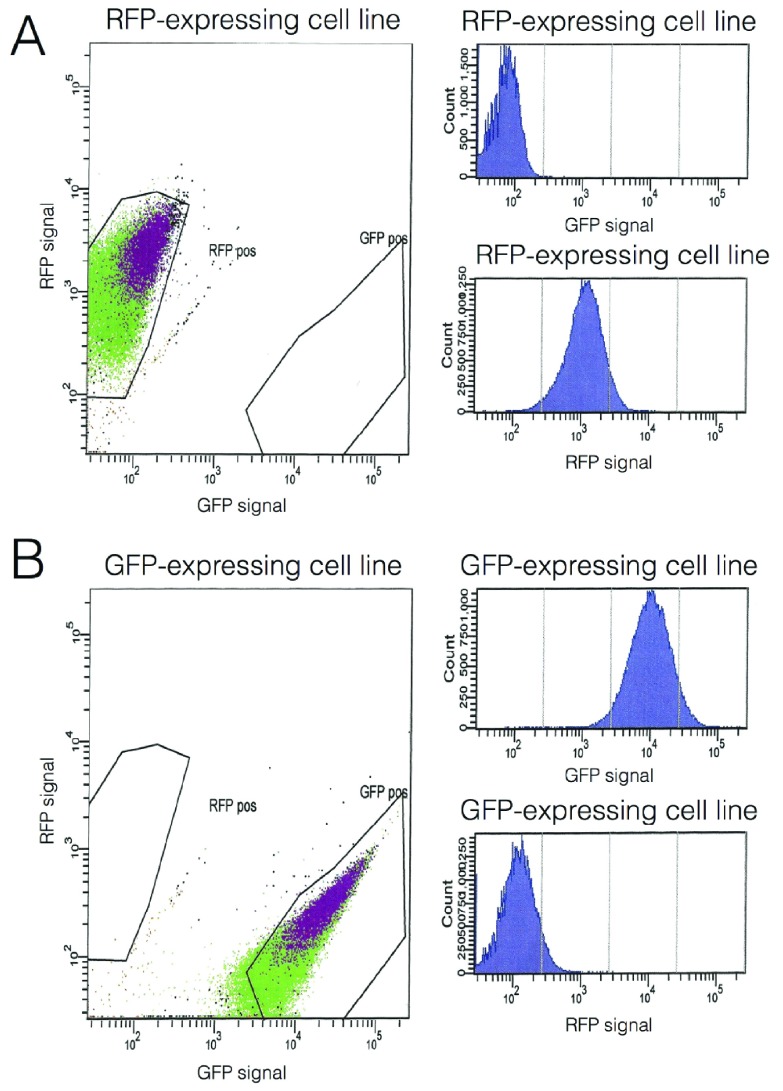
Flow cytometry analysis of fluorescent reporter cell lines. RFP and GFP expressing DH1 cell lines were analyzed by flow cytometry to confirm that cell lines are homogenously fluorescent, which confirms the validity of our approach. 10 ^6^ exponentially growing cells of each cell line were analyzed via excitation by an Argon laser at 488nm and detection with emission filter sets specific to RFP and GFP. Excitation and emission settings were kept constant throughout analysis. **A**) RFP-expressing cells display a unimodal fluorescence in the RFP channel (bottom right plot), and background-level fluorescence in the GFP channel (upper right plot). The GFP vs RFP plot (left) displays a fluorescently homogeneous population in the RFP-specific analysis gate. **B**) GFP-expressing cells display the reciprocal features. Importantly, less than 1% of cells were not fluorescent, ruling out artifacts in our cell counting approach.

Cells in different nutritional states have different aggregation rates on their own. We next examined how cells in different nutritional states interact in mixtures in order to analyze how introducing population nutritional state heterogeneity affects population partitioning. Pairwise mixtures of FM-grown cells with HL5-grown cells and NS-grown cells with NS Glu-grown cells were tested. Cells grown in NS or NS Glu that did not differ when alone showed no difference in behavior when in mixtures ( Supplementary Figure S4) (F=1.54, p=0.27). On the other hand cells grown in FM were enriched 3 times more in the non-aggregating cell fraction when in mixture with HL5-grown cells, 15.4±7.12%, than on their own, 5.85% ( Figure 2C). HL5-grown cells aggregated equally well when in mixture with FM-grown cells or not. As a control we monitored contribution to spores for both mixtures. As previously shown, cells grown in rich medium were enriched in spores in both NS Glu:NS and HL5:FM mixtures ( Supplementary Figure S5).

We conclude that nutritional state distinguishes non-aggregating cells from aggregating cells, with better fed cells aggregating more efficiently, and that interactions between cells according to their nutritional state biases further partitioning between aggregating and non-aggregating cell fates. Moreover, the 1:1 mixed population of cells having different nutritional status showed a higher fraction of non-aggregating cells than the average of both populations. This is consistent with our data obtained with populations grown on heterogeneous food source showing a higher proportion of non-aggregating cells. Cells grown on low nutrient medium have higher chances of becoming non-aggregated cells than cells grown on rich medium. The fact that NS-grown cells displayed the same behavior as NS Glu-grown and HL5-grown cells is probably because cells were relatively well fed in all three cases and not much affected by the absence of glucose ^16^. On the other hand FM-grown cells showed smaller cell size, slower growth and lower inner cell density indicating that they were affected by growth in poor medium (our unpublished observation). We can speculate that poorly fed FM-grown cells have low energy reserves, and that they consequently invest less into energetically costly multicellular development and thus aggregate less. The fact that, in mixtures with HL5-grown cells, FM-grown cells showed an even lower rate of aggregation indicates the effect of cell-cell interactions during aggregation. No difference in the timing of aggregation was seen between FM- and HL5-grown cells. Therefore, cell nutritional state rather than aggregation timing was the cause of the differences in the fraction of non-aggregating cells.

### Genetic factors affect population partitioning

After exploring nutritional state effects, we tested whether different genetic backgrounds can lead to different population partitioning. In Figure 3A we show that two axenic strains, DH1 and AX3, significantly differ in the fraction of non-aggregating cells (p=0.0008). The DH1 strain showed 13.4%±2.8% of non-aggregating cells, which is five times higher than for the AX3 strain (2.5%±0.6%). This shows that the non-aggregating cell fraction depends on the genetic background and varies significantly between axenic wild-type strains.

Following these results, we explored which genetic factors may affect the cell propensity for aggregating or non-aggregating fates. For this, we first tested strains with single gene mutations in aggregation pathways. We used two mutants defective in signal sensing: 1) *carA,* a mutant in cAMP receptor protein cAR1, which is essential for binding the chemo-attractant cAMP and 2) *pdsA*, a mutant in cAMP-phosphodiesterase (PDE), which removes cAMP from its cAR1 receptor making it sensitive again to the aggregation signal ^10^. Our results confirmed the previously reported result that when plated on nutrient-free agar, both strains showed no aggregation at all ( Figure 3A) ^28, 29^. This shows how single gene mutations may have a drastic effect on population partitioning. It is known that the presence of wild-type cells can rescue the non-aggregating *pdsA* phenotype (non-cell autonomous) ^30^. Our technique allows the quantification of aggregation efficiency of mutant and wild-type cells in mixtures. We thus varied the ratio of wild-type cells (AX3 or DH1) in mixtures with mutant *pdsA* cells from 10% to 90% and quantified how it affects aggregation of *pdsA* mutant and wild type strains. For both DH1: *pdsA* and AX3: *pdsA* mixtures, increasing the ratio of wild type cells decreased the proportion of *pdsA* non-aggregating cells ( Figure 3B–D). Aggregation rescue of mutant cells came at a cost for the DH1 strain; the fraction of non-aggregating cells for DH1 increased in mixtures with *pdsA* ( Figure 3C, D). In AX3: *pdsA* mixtures, AX3 cells aggregated as much as when on their own and *pdsA* cells aggregated more than when on their own ( Figure 3B, D), suggesting that AX3 produces more PDE protein than DH1. More generally, we propose that expression levels of cAMP-phosphodiesterase may tune the non-aggregated cell fraction. Low concentration of cAMP-phosphodiesterase would increase the fraction of non-aggregating cells.

We found that differences in starvation sensing affect the partitioning between aggregating and non-aggregating fractions ( Figure 2A). The *phg2* mutant strain has been shown to have early onset of starvation compared to its parental strain DH1 due to a higher nutrient starvation sensing threshold ^31^. We used this single gene mutant to test the effect of the nutrition starvation sensing threshold on partitioning. In addition, the *phg2* gene codes for a serine/threonine kinase regulating cell substrate adhesion, actin cytoskeleton organization and motility ^32^. When tested alone, *phg2* produced a similar fraction of non-aggregated cells when compared to its parental strain DH1, 12.6%±4.3% (p=0.7). We further tested the behavior of *phg2* in 1:1 mixtures with wild-type strains DH1 and AX3. Mixing at 1:1 led to an increase of the non-aggregating cell fraction for *phg2* and its DH1 parent ( Figure 3F, G), while AX3 aggregated equally well as when on its own ( Figure 3E, G). This once more demonstrates that in mixtures, strains mutually affect each other’s non-aggregating cell fractions. Indeed, the *phg2* mutant aggregates less in 1:1 mixtures with wild-type cells than on its own, even in mixtures with its parent DH1 wild-type strain that has a similar aggregation fraction on its own. Moreover, in 1:1 mixtures of *phg2* with DH1 or AX3, the global mixed population shows a significant increase in the fraction of non-aggregating cells with respect to both pure populations. This is again reminiscent of our previous results that population heterogeneities in terms of nutritional state (cells grown on heterogeneous bacterial lawns, or on mixtures of cells grown on HL5 vs. FM) increase the non-aggregation fraction of the global population. In addition to starvation sensing, the dysfunctional cytoskeleton organization and motility of the *phg2* strain could explain the lower propensity of *phg2* cells for aggregation.

Overall, genotypes determine the non-aggregating cell fraction in isogenic populations, and different genotypes affect each other’s non-aggregating cell fraction in non-isogenic populations, with an apparent increase in the global non-aggregating cell fraction with respect to the corresponding isogenic populations.

### Cell history and cell fate

We further tested whether non-aggregation is due to a stable mutation or a stochastic switch affecting aggregation vs non-aggregation. Can the same population partitioning be reproduced by starting from only aggregating or only non-aggregating cells? Answering this question allows us to: i) rule out any stable genetic differences between aggregating and non-aggregating cells (while a genetic switch remains a possibility) and ii) examine the inheritance of cell fate. When non-aggregating cells are *de novo* fed with bacteria, they resume growth on new nutrients (see below) until they are exhausted and finally aggregate upon starvation ( Figure 4A–C and Movie S2). This shows that non-aggregating cells are not mutant cells that cannot aggregate, but rather cells that are not responding to the aggregation signal at a given time point. Further on in Figure 4D we show that a population of germinated spore cells dividing 3 to 5 times upon germination partitions into aggregating and non-aggregating cells with the same fractions as a population of exponentially growing cells. This demonstrates the strong persistence of population partitioning and the fast loss of cell fate memory. Molecular mechanisms involved in this process may be either an epigenetic or a genetic switch.

### Individual-level costs and benefits of the non-aggregating cell fraction

We have shown that upon starvation *D. discoideum* cell populations partition into cells that aggregate and cells that do not aggregate, and that non-genetic and genetic cell characteristics affect cell fates. We next analyze evolutionary consequences of this population partitioning. To do this we analyzed fitness costs and benefits of both phenotypes on individual and population levels.

**Figure 5.**
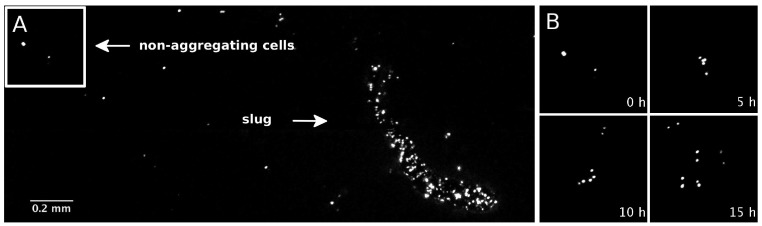
Non-aggregating cells grow on new incoming nutrients. 18h after plating cells on nutrient-free agar, aggregating cells have formed slugs while non-aggregating cells are starving. Fresh nutrients (dead bacteria) were added at this point. **A**) Red fluorescence image of slugs and non-aggregating cells at the time of new nutrient supply. **B**) Inset from A showing a non-aggregating cell that resumes dividing over time upon addition of new nutrients (the number of red dots increases over time as non-aggregating cells divide). Non-aggregating cells are capable of resuming growth immediately upon food arrival while aggregating cells are embedded in development.

Once in an aggregate a cell is irreversibly committed to the multicellular development program ^14^. During the 24h duration of development, cells cannot divide even if nutrients become available. Therefore, if food becomes available during the developmental period, non-aggregating cells may have an advantage over aggregating cells by immediately resuming growth. We tested this by adding a bacterial suspension to a starving *D. discoideum* population during the course of development. At this point aggregates were at the slug stage and non-aggregating cells in their vicinity had direct access to food ( Figure 5A). In Figure 5B and Movie S3 we show that non-aggregating cells are capable of resuming cell division directly after arrival of nutrients, while slugs (formed of non-dividing aggregated cells) continue moving through the bacterial lawn and form fruiting bodies. Our observation is clearly distinct from previous reports describing the dedifferentiation and re-growth of cells from artificially disaggregated slugs put in contact with fresh nutrients ^33^ since non-aggregating cells do not originate from slugs and are therefore not differentiated into prespore or prestalk. We also observed that by the time fruiting bodies are formed, non-aggregating cells have already consumed a high amount of nutrients, which will probably affect spore fitness by limiting the resources available for spore germination and proliferation (Movie S3).

Non-aggregating cells are motile and do not seem to enter a dormant state like spores do, making them likely to be much less fit than spores during prolonged starvation. Previous studies have reported starvation-induced mortality curves showing that most cells survive for 4 to 7 days ^20^ (corroborated by our unpublished results). These studies demonstrated that, in the absence of food, cells survive through autophagy, degrading their own cytoplasmic components and organelles. Once cells have degraded most of the inner cell components and autophagy can no longer serve as a mode of survival, mortality rate increases and cells die within a day. Non-aggregating cells are expected to pay the same survival costs during long starvation periods.

### Model: evolutionary framework

To test how phenotypic partitioning affects population fitness, we developed a mathematical model that mimics the *D. discoideum* life cycle. We asked whether particular non-aggregation rates are selected in fluctuating environments having different, constant or variable, starvation duration and frequency. The model was defined as follows. Not all cells aggregate ( Figure 1), cells that do not aggregate die at a defined mortality rate ^20^ (and our unpublished results), non-aggregating cells are capable of resuming growth upon arrival of bacteria ( Figure 5A and B, Movie S3); once in an aggregate cells do not divide and are committed to multicellular development until the end ^14^. All the parameters used in the model, such as growth rate, sporulation efficiency and germination efficiency were measured experimentally (see Supplementary materials). Since aggregation is an adaptation to starvation and since the duration of starvation affects costs and benefits of each phenotype (mortality, growth), we tested how the duration of starvation determines the optimal non-aggregating rate.

**Figure 6.**
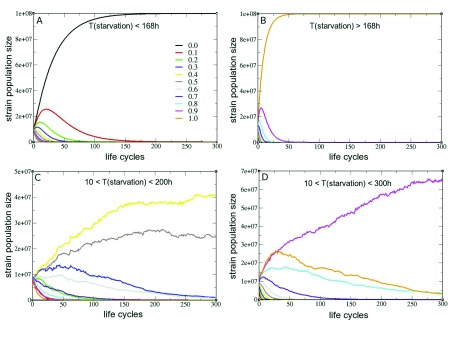
Population partitioning is advantageous in fluctuating environments. Our numerical model involves eleven strains with different fixed investments into non-aggregating cells that were competed under different starvation conditions. Strain investment into non-aggregating cells varies from 0 to 1, with 1 corresponding to complete aggregation and 0 to no aggregation. The duration of the starvation period was varied from <168h ( **A**), >168h ( **B**), randomly taken between 10h and 200h ( **C**), randomly taken between 10h and 300h ( **D**). For systematically long (>168h, **B**) and short (<168h, **A**) durations of starvation, strains with 100% aggregation and 0% aggregation take over respectively. For random starvation duration, a particular aggregation rate is selected, for instance 0.4 for 10h<T<200h ( **C**) and 0.9 for 10h<T<300h ( **D**), and thus the superimposition of both strategies is the optimal response.

We defined 11 strains differing in their non-aggregating cell fractions and calculated their geometric growth rate as a fitness measure. Investment into non-aggregating cells ranged from all cells aggregate (value 1) to none of the cells aggregate (value 0) and was fixed for each strain during the whole competition. For the sake of simplicity, we did not take into account interactions between strains that may increase or decrease aggregation rates, even though our experimental results demonstrated that such interactions do occur and that heterogeneities play a role. In Figure 6A and B we show that under constant starvation periods there are two stable strategies: no aggregation for starvation periods under seven days (168h), and complete aggregation for longer starvation periods. The switch point at 168h is due to the 100% mortality rate after this period. Use of different mortality rates and functions did not significantly change the results (the time period for each optimal strategy just shifted). Since natural environments are rarely constant, with only long or only short starvation periods, we tested competition in environments with fluctuating, long (>168h) and short (<168h) starvation periods. We find that population partitioning into both aggregating and non-aggregating cells gives the highest (geometric) fitness benefits in these fluctuating conditions ( Figure 6C and 6D). The results also show that different fluctuations in starvation duration select for different non-aggregating rates. This is in agreement with other models and experiments that showed that optimal population response depends on the rate of environmental fluctuations ^2, 3, 34^.

## Discussion

We report that upon starvation stress a population of *D. discoideum* amoebae partitions into the widely studied multicellular structures (consisting of live but dormant spores and dead stalk cells) and a fraction that remains unicellular (non-aggregating cells). We have measured the fraction of non-aggregating cells and found that it can amount to up to 15% of the total population in realistic starvation conditions. This is much higher than the 2–3% of non-aggregating cells that result in the standard sudden starvation protocols, and shows that it is important to mimic natural conditions. Non-aggregating cells are live (Supplementary Movie S1), non-stably mutated cells ( Figure 4) that occur in both axenic strain and natural isolates ( Supplementary Figure S2). We have thus demonstrated that the non-aggregating cell fraction in natural starvation conditions constitutes a significant component of the population-level starvation response, at least of the order of the stalk cell subpopulation. For our detailed analysis of genetic and non-genetic contributions, we have nevertheless employed the standard sudden starvation protocol to ensure full control over cell population composition and nutritional state, even though this protocol tends to minimize the non-aggregating cell fraction.

In isogenic populations, we show that partitioning depends on phenotypic heterogeneities linked to cell nutritional state. This is a previously reported determinant of the differentiation between spore and stalk cell fate in aggregates ^27^, together with intracellular Ca ^2+^ levels ^35^ and cell cycle phase ^36^. Decreased aggregation in cells with low nutritional status correlates with lower investment into energetically costly aggregation. The nutritional state-dependent partitioning of the social amoebae population is reminiscent of previous studies reporting non-genetic population heterogeneities in *Escherichia coli* persistor strains ^37^, *Pseudomonas fluorescens* colony morphology ^38^, *Bacillus subtilis* sporulation ^9^ and many others.

Different genetic backgrounds can give rise to different levels of heterogeneity ^9, 15, 26, 39^, giving insights into the underlying molecular mechanisms. We demonstrate that genetically different wild-type strains show different non-aggregating cell fractions. This has important implications when drawing a parallel with natural conditions. Distinct genetic strains in nature may show different aggregation fractions leading to competition between different aggregation strategies, as we explore in our model in Figure 6. Further, our results on single-gene mutants underlie possible mechanistic differences between aggregated and non-aggregated cells. We propose that genetic factors that regulate the timing of starvation, signal sensing efficiency and aggregation efficiency largely determine whether a cell adopts the aggregating or non-aggregating phenotype. We confirm that *cAR1* and *pdsA* mutants ( Figure 3A and 3B), which are deficient in signal sensing, clearly display non-aggregating cell fractions that differ from their parent strain. Differences in gene expression levels are a known source of phenotypic heterogeneities; *comK* in *B. subtilis* cell competence ^40^, *spoA* in *B. subtilis* sporulation ^9^, *Saccharomyces cerevisiae* FLO-dependent phenotype ^41^. It would be very interesting to monitor the same for early developmental genes, expressed at the beginning of aggregation, to see if distinct expression levels correlate with aggregating and non-aggregating cell fates. Genes that control the efficiency of aggregation such as *cAR1* and *pdsA* are potential candidates.

Our results on interactions between mutant and wild type cells in mixtures show that partitioning of social amoebae populations is a complex process, and that competition between genotypes with different aggregation rates is non-linear. In other words, the behavior of strains in mixtures is not the mere linear superposition of their behaviors when on their own, which is reminiscent of the well-documented behavior of strains in mixtures during sporulation experiments ^42– 44^. Importantly, even if certain mutants such as *phg2* (starvation sensing and motility mutant) do not display a fraction of non-aggregating cells that differs from their parent strain, the non-aggregating cell fraction of the global population may increase ( Figure 3) as a result of heterogeneities as is the case of cells grown in heterogeneous conditions ( Figure 2, Supplementary Figure S3). We propose that population heterogeneities, due to both genetic and phenotypic causes, play a key quantitative role in population partitioning between unicellular and multicellular cell fates. The effects of nutrition status heterogeneities we report are reminiscent of the previously reported link between nutrition status, cell cycle or Ca ^2+^ content heterogeneities and prespore vs. prestalk differentiation. In nature, social amoebae gradually deplete their food source and spatial distributions of genetic clones largely overlap, thus making both phenotypic and genetic heterogeneities realistic features of the unicellular vs. multicellular starvation response we describe, and hence reinforcing the ecological significance of our findings.

Different phenotypes are often associated with different fitness costs and benefits. In our case, dormant spores survive for months without nutrients but take advantage of incoming food with a delay in comparison to non-aggregating cells. This lag corresponds to the duration of multicellular development and germination, up to 30h or 8 times the single cell division time. Therefore, non-aggregating cells may divide up to 8 times when nutrients are present soon after the beginning of multicellular development, while aggregating sporulating cells do not divide until the end of germination ( Figure 5). This confers a considerable evolutionary advantage to non-aggregating cells in such situations (2 ^8^=256-fold). Our model explores the long term, evolutionary consequences of these effects on the competition between clones with different aggregation rates in fluctuating environments. We find that the aggregation rate is under selection in fluctuating environments and that the optimal rate depends on fluctuations in starvation duration and frequency.

Strategies in which different phenotypes may show differential fitness advantages in different environments are often called bet hedging, and have been shown to be adaptive in fluctuating environments ^2– 6, 38^. In plants, the success of germination often depends on precipitation. Since rainfall is unpredictable and variable, the diversification of germination timings within season was predicted and demonstrated ^7^. Similar examples include mosquito egg hatching ^45^, copepod egg diapause ^46^, phenotypic switching in *S. cerevisiae* ^3^, persistor phenotype in *E. coli* ^34^ and many others ^7^. *B. subtilis* behavior has the greatest resemblance to what we report in *D. discoideum*. Upon starvation the population of *B. subtilis* partitions into sporulating and non-sporulating cells. Non-sporulating vegetative cells postpone their sporulation by consuming secondary metabolites and cannibalizing each other, and have the advantage of immediate growth upon arrival of nutrients ^9, 47^. In *D. discoideum* aggregation is required for sporulation. Since sporulation is beneficial only if the duration of starvation is long enough ( Figure 6), and since cells cannot *a priori* sense the duration of starvation, population diversification should be the optimal response. This is exactly what we get with our model in Figure 6. We therefore propose the hypothesis that partitioning between non-aggregating and aggregating cells is a form of bet hedging in environments with unpredictable durations of starvation. Bet hedging behaviors result from switching between different phenotypes. Consistently with our hypothesis, we show that a population of only aggregating (spores) or only non-aggregating cells re-partitions into aggregating and non-aggregating cell fates upon starvation following re-growth for a couple of cell divisions.

Consequently, our results have implications for studies of cooperation that use social amoebae as a model system. Studies on mixtures of non-isogenic cells show that some genetic clones bias their ratio into spores ^48, 49^. However, the behavior of a mixture of more than two clones going through a series of growth and sporulation cycles cannot be entirely explained based on biases observed in pairwise mixtures during one round of sporulation ^50^. The whole life cycle needs to be taken into account, as competition occurs between strains not only during sporulation within aggregates but also at other steps such as unicellular growth, with complex trade-offs ^51, 52^. Here we characterize, in this respect, the aggregation step of the life cycle, and show that the previously neglected non-aggregating cell fraction constitutes a significant component of the population-level starvation response. This fraction is different for different genetic clones, it is at least of the order of the stalk cell subpopulation and interactions between clones do affect this fraction. Therefore, this additional unicellular cell fate needs to be taken into account when defining a clone’s behavior when alone and in mixtures. We propose to characterize amoebae behavior not only with respect to spore vs. stalk investment in aggregates but also with respect to aggregation vs. non-aggregation investment. This means that instead of classifying phenotypes as just stalk-biased and spore-biased we may find a much richer repertoire, involving high aggregation efficiency but low investment into stalk, low aggregation efficiency and high investment into stalk, and so forth.

Population partitioning can also be interpreted as probabilistic expression of ‘social behavior’ (here, aggregation). Genetic and non-genetic mechanisms may regulate the probability of a cell acquiring a social/aggregating phenotype. It has been shown that such probabilistic expressions of social phenotype may be strategies that play an important role in stabilizing ‘cooperation’ ^53, 54^. The results presented here reinforce the notion that one should allow individuals to ‘opt out’ of a social interaction to gain a more complete understanding, as has been argued for some time by game theoreticians ^55^. For instance, allowing individuals to opt out of a social interaction may lead to evolutionary cycles ^53, 56, 57^. Our results show that environmental stochasticity affecting relative fitness of social and asocial individuals may also favor opting out of at least a part of the population. It will be important to investigate further the role of population partitioning into aggregating/social and non-aggregating/asocial phenotypes on the stabilization of cooperation.

Overall, we have demonstrated that the nutritional stress response of the social amoebae *Dictyostelium* consists of the coexistence of a unicellular non-sporulating strategy and a multicellular sporulating strategy. We provide evidence that cell fate is determined by four types of factors: (i) autonomous, linked to cell genotype, (ii) environmental, (iii) dependent on gene × environment interactions, and (iv) dependent on cell-cell interactions. We propose the hypothesis that social amoebae thus lie at the intersection of two key concepts in evolutionary microbiology, namely cooperation and bet hedging, and define a unique model system to explore this new frontier.

## Data availability

figshare: Aggregation vs. nonaggregation strategies in *Dictyostelium discoideum* amoebae in response to starvation stress: raw data, doi: 10.6084/m9.figshare.1052997 ^58^

## Supplemental experimental protocols

### Germination efficiency

Spores were plated on 9cm SM/5 plates with 200μl of overnight *K. aerogenes* culture. Spores germinated, cells divided, consumed bacterial food and when starved formed new spores. Fresh spores were re-suspended in ice-cold KK2 buffer with 0.1% Tween20 (SIGMA and vortexed. 100 spores were plated with 500μl of overnight *K. aerogenes* culture and plated on 14cm petri dish with SM/5 agar. After 3 days we counted number of formed plaques. Germination efficiency was counted as N _spores_/N _plaques_. Experiment was done for 6 wild type strains; 34.1, 28.1, 105.1, 63.2, 85.2 and 98.1 isolated from North Carolina ^59^. Experiment was repeated 7–9 times with 3 replicas per measurement. The mean of the 6 strains was taken as the value for germination efficiency in the model.

### Growth rate on bacterial plates

Spores were plated on SM/5 plates with 200μL of overnight *K. aerogenes* culture. Spores germinated into cells and cells started dividing. 15–20h after plating spores cells were removed from the plates by washing the plates in ice-cold KK2 buffer. The cell suspension was centrifuged 3 times in ice-cold KK2 for 5min, 300g to remove bacteria so we could measure the cell concentration. 1×10 ^5^ cells were re-suspended in 500μL of overnight *K. aerogenes* and plated on 15cm petri dish with SM/5 agar. 16–20h after plating we started to measure cell growth. Growth was measured for 3 independent plates/time point every 2h during 8h. For each measurement cells were removed by scraping the cells from the plate in ice-cold KK2 buffer to prevent cell division. The cell suspension was centrifuged 3 times for 5min, 300 g on 4°C to remove bacteria. Cells were counted using a haemocytometer. The growth curve was represented as log2 of the cell number over time. The growth rate was calculated as the slope of the linear regression of the log2 growth curve. The experiment was done for 6 wild type strains; 34.1, 28.1, 105.1, 63.2, 85.2 and 98.1 isolated from North Carolina ^58^. The mean of the 6 strains was taken as the value of growth rate in the model.
