# Supplementary material for: An evolutionarily significant unicellular strategy in response to starvation in Dictyostelium social amoebae
Source: F1000Res. 2014 Dec 5;3:133. Originally published 2014 Jun 24. [Version 2] doi: 10.12688/f1000research.4218.2 (PMC4184345; doi:10.12688/f1000research.4218.2)
Supplement: Peer review history

# Peer review: Referee response for version 2

**DOI:** 10.5256/f1000research.5915.r5222

**Published:** 2014-12-05

**Authors:** Paul Rainey

**Affiliations:** 1 NZ Institute For Advanced Study, Massey University, North Shore City, New Zealand

I approve the authors' revision.

I have read this submission. I believe that I have an appropriate level of expertise to confirm that it is of an acceptable scientific standard.

# Peer review: Referee response for version 1

**DOI:** 10.5256/f1000research.4516.r5848

**Published:** 2014-08-18

**Authors:** Paul Rainey

**Affiliations:** 1 NZ Institute For Advanced Study, Massey University, North Shore City, New Zealand

This is an important paper that draws attention to non-aggregating cells of Dictyostelium and shows that under nutrient deprived conditions such cells arise by a stochastic mechanism and when considered alongside cells that commit to slug – and ultimately spore – formation appear to represent a bet-hedging strategy. Such a strategy may be adaptive in the face of uncertainty surrounding unpredictable fluctuations in resource availability. This little considered aspect of the biology of Dicty has significant implications for how we think about the function of this amoeba in its natural environment.

The article content is of good quality and the experiments are well conducted. However, I suggest re-writing the section entitled “ *Genetics of population partitioning*…” to bring greater clarity. 

Experiments showing that loners can switch back to spore-formers do not demonstrate that the switch is epigenetic (the switch could be mutational (a genetic switch)). Similarly, the hypothesis of bet hedging is not tested: it is an entirely reasonable hypothesis, but not proven.

There is need to provide experimental details in the figure captions concerning number of replicates, nature of error and statistical analyses (where appropriate).

Specific suggestions

**Abstract**

- *“...studies of microbial cooperation...”*
  I recommend deleting this.
- *“Non-aggregating cells have an advantage over cells in aggregates since they resume growth earlier upon arrival of new nutrients, but have a shorter lifespan under prolonged starvation.”*
  Compared to what? Compared to spores?
- *“We find that phenotypic heterogeneities linked to cell nutritional state bias the representation of cells in the aggregating vs. non-aggregating fractions, and thus regulate population partitioning.”*
  I probably wouldn't use the word regulate. Maybe just 'affect'
- *“D.discoideum thus constitutes a model system lying at the intersection of microbial cooperation and bet hedging, defining a new frontier in microbiology  and evolution studies”*
  This is not such a great sentence. Rather than try and sell it in this way, I would recommend that the authors either delete the sentence, or emphasize that they have drawn attention to an overlooked aspect of the biology of Dicty that may have ecological relevance. 

 

**Introduction**

- *“Yet, living environments typically deviate from these conditions.”*
  Remove “ *living*”
- *“Our main motivation was to study a previously known but neglected fact that not all cells aggregate upon starvation.”*
  Add reference
- Remove “ *While often considered an experimental error or just insignificant*”
- *“We asked whether the fraction of non-aggregating cells constitutes an important component of the adaptive response to stress.”*
  I thought the main idea you are testing is the possibility that solitary cells constitute a bet hedging strategy that may have adaptive significance in the face of unpredictable changes in the nutritional status of the environment?

 

**Materials and methods**

Model

- *“The advantage that non-aggregating cells have is a head start when conditions improve, as spores produced by aggregating cells need time to develop.”*
  The advantage may come even earlier than spore formation. Is the commitment to form fruit bodies terminal? If so, then right from the outset, there is likely to be an advantage for spreading risk. In many ways this is very similar to what has been worked out for *Bacillus* and the idea that the commitment to sporulation is delayed as long as possible (see work from Losick's group).

**Results**

- *“...the observation that non-aggregating cells are actively moving, live cells that are intermixed with aggregating cells at the onset of starvation”*
  Remove the comma

Phenotypic plasticity affects population partitioning

- *“While stationary phase cells show no significant difference compared to exponentially growing cells, cells feeding on a homogenous bacterial lawn and thus gradually starving showed a 3-fold increase in the proportion of non-aggregating cells,6.3* *±3.17% (p=0.027).”*
  Perhaps mention in the results what a homogeneous vs. heterogeneous lawn of bacteria means.
- *“Four different media were tested: HL5 rich medium, FM minimal medium, NS with 85mM glucose (NS Glu) and NS medium.”*
  Without going to the M&M I don't know how to read the differences between the media. Why did you not systematically change C / N and the ratio of C:N?
- *“We conclude that nutritional state distinguishes non-aggregating cells from aggregating cells, and that interactions between cells according to their nutritional state biases further partitioning between aggregating and non-aggregation cell fates.”*
  But what is it about the nutritional status of the media?

Figure 2

- Axes – should Homog. and Heterog. lawn instead of loan.
- Legend - Mean and standard deviation of *x* replicates (state number of replicates).  State the meaning of the lines.  Also, was a posteriori test was applied (assuming the lines are indicating the result of this analysis).  If this was ANOVA first and then a posteriori test, then state the ANOVA result as well (without this I don't know what the p levels mean).

Figure 3

- Legend - See comments above regarding representation of statistical results.

Genetics of population partitioning into aggregating and non-aggregating fractions

 

- This section is unclear to me. It is not what I would call "genetics". I am not sure to what extent this is an *'exploration of the genetic mechanisms affecting aggregating / non-agg fates*'. I understand that the authors have taken two mutants that can be rescued when grown with wild type. I really don't understand what these experiments tell us. This should be clarified and made more explicit.

 

Cell history and cell fate

- Change *“...bet hedging-like strategy between...” *to *“stochastic switch affecting”*
- *“Answering this question allows us to: i) rule out any genetic differences between aggregating and non-aggregating cells and ii) examine the effect of epigenetic inheritance of cell fate.”*
  This does not rule out the possibility of a genetic mechanism. For example, a genetic switch could be responsible.
- *“This demonstrates the strong persistence of population partitioning and the fast loss of cell epigenetic memory.”*
  Or a genetic switch. I think this shows that the solitary types are not mutants. I do not think this shows evidence for an epigenetic switch.

Figure 4

- Legend - No. of replicates? Nature of error bar? etc.

Model

- The model is useful and shows nicely that solitary cells and slug / spore forming cells have likely ecological relevance. One thing missing is any parameter to describe interactions among cells in the slugs / fruit body (and between different genotypes) that are likely to be important components of fitness, but perhaps at this stage there is insufficient empirical data for this to be usefully attempted. 

Figure 6

- I think you should make clear in the caption that these are results from a mathematical model.

**Discussion**

- *“We report that upon starvation stress a population of D. discoideum amoebae partitions...”*
  I think you should avoid the term *“stress”*. It is meaningless.
- *“For our detailed analysis of genetic and non-genetic contributions...”*
  The analyses are not particularly *“detailed”*. I suggest removing this.
- *“Bet hedging behaviors result from epigenetic switching between different phenotypes.”*
  Not necessarily epigenetic. Take contingency loci in pathogenic bacteria for example.
- *“This demonstrates the epigenetic bet hedging-like nature of population partitioning in D. discoideum.”*
  No. It demonstrates neither epigenetic, nor that the strategy is a bet hedging one. The behaviour is consistent with a bet hedging strategy. 
- *“This means that instead of classifying phenotypes as just altruistic and cheaters we may find a much richer repertoire, involving social cheaters (high aggregation efficiency but low investment into stalk), asocial altruists (low aggregation efficiency and high investment into stalk), asocial cheaters (low aggregation efficiency and low investment in the stalk) and so forth.”*
  I would suggest you could also put aside the anthropomorphic language and refer to solitary cells and slug / spore forming cells and their interactions.
- *“Population partitioning can also be interpreted as probabilistic expression of social behavior.”*
  See previous comment. 

I have read this submission. I believe that I have an appropriate level of expertise to confirm that it is of an acceptable scientific standard.

# Peer review: Referee response for version 1

**DOI:** 10.5256/f1000research.4516.r5221

**Published:** 2014-07-04

**Authors:** Richard Gomer

**Affiliations:** 1 Department of Biology, Texas A&M University, College Station, TX, USA

This is a very nice explanation of why some Dictyostelium cells do not join into aggregates when the population of cells starves. Many of us have observed this phenomenon, and wondered why this happens. The explanation is that if nutrients suddenly appear while aggregated cells are undergoing development, the non-aggregating cells can immediately begin growth and proliferation, while the aggregated cells have to plod through development and then spore germination before they can start dividing. The authors show both data as well as nice mathematical models of this bet-hedging strategy.

One minor correction - in Figure 1A 'loan' should be 'lawn'. 

In the future, finding the mechanism that causes a small percentage of cells to not aggregate may shed light into new mechanisms of cell population symmetry-breaking and differentiation.

I have read this submission. I believe that I have an appropriate level of expertise to confirm that it is of an acceptable scientific standard.
